# 281. Mathematical modeling of corticosteroid induced secondary infection and In silico Dose exploration of Dexamethasone, Hydrocortisone

**DOI:** 10.1093/ofid/ofad500.353

**Published:** 2023-11-27

**Authors:** Dong-Hyun Kim, Eun Kyuong Chung

**Affiliations:** Kyung Hee University, Seoul, Seoul-t'ukpyolsi, Republic of Korea; Kyung Hee University, Seoul, Seoul-t'ukpyolsi, Republic of Korea

## Abstract

**Background:**

The use of steroids in infected patients is necessary to modulate the inflammatory response that is triggered by infection. However, there is a concern that steroid use could increase the risk of secondary infection. Here, To quantitatively and mechanistically evaluate the risk of secondary infection, we simulate different types and doses of steroids by utilizing a state-of-the-art hybrid PBPK-QSP modeling approach.

**Methods:**

A minimal PBPK model of vancomycin and dexamethasone/hydrocortisone was developed and combined with a mathematical QSP model of bacterial infection and immune response. The models were linked to each other in R 4.2.2 using the rxode2 package. Usual dosing regimen of vancomycin and steroids were simulated, and long-term use of high-dose steroid therapy with vancomycin was also simulated. Vancomycin dosing was fixed at 1500mg IV infusion q12h for 7 days, dexamethasone 5 mg or 70 mg q12h IV infusion for 7 days, and hydrocortisone 100 mg or 500 mg q12h IV infusion for 7 days, with 1.2 times of the maintenance dose given as a loading dose. Plasma and lung PK profiles were derived using the minimal PBPK model to be combined seamlessly with the QSP model. Model-based simulations were conducted over a period of 1000 hours.

**Results:**

Our minimal PBPK model simulated the PK profiles of vancomycin as well as dexamethasone and hydrocortisone in plasma and lung within the physiologically plausible range (**Figure 1**). In the case of dexamethasone 70 mg, the number of neutrophils decreased by about half compared to the case of dexamethasone 5 mg, and the number of bacteria in the lung compartment substantially increased (13.6% of the initial inoculum) after 300 hours (**Figure 2**). The other regimens did not show a significant increase in the number of bacteria.

**Figure 1.**
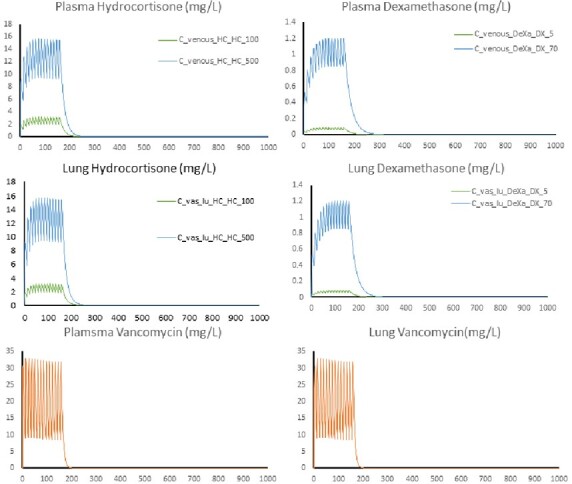
The Plasma and PK profiles of Dexamethasone, Hydrocortisone and Vancomycin by using PBPK models.

**Figure 2.**
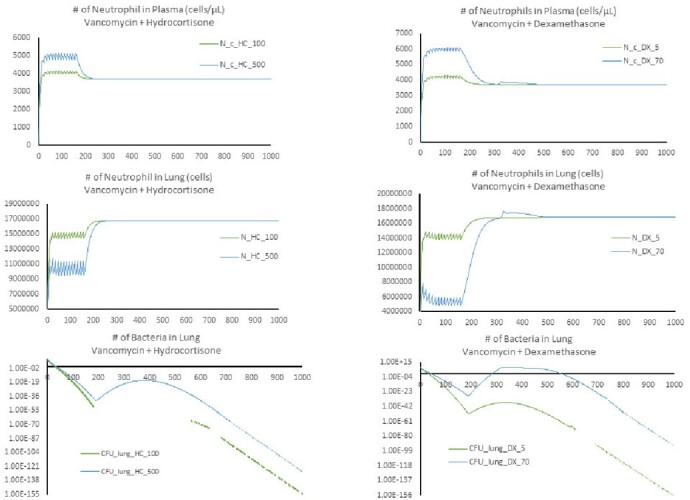
In silico simulation results of Bacterial infection and immune response during concomitant use of vancomycin and steroids.

**Conclusion:**

Our modeling results suggest that the usual dose of steroid commonly used in infected patients does not increase the risk of secondary infection. However, long-term use of high-dose steroid therapy could increase the risk of secondary infection. When long-term use of high-dose steroid therapy is needed, Hydrocortisone could be a better option than dexamethasone from the perspective of infection risk

**Disclosures:**

**All Authors**: No reported disclosures

